# Methylation analysis and HPV genotyping of self-collected cervical samples from women not responding to screening invitation and review of the literature

**DOI:** 10.1371/journal.pone.0172226

**Published:** 2017-03-06

**Authors:** Annarosa Del Mistro, Helena Frayle, Martina Rizzi, Gianpiero Fantin, Antonio Ferro, Paolo Matteo Angeletti, Paolo Giorgi Rossi, Emma Altobelli

**Affiliations:** 1 Immunology and Diagnostic Molecular Oncology Unit, Veneto Institute of Oncology IOV IRCCS, Padua, Italy; 2 Maternal and Child Department, Local Health Unit 7, Pieve di Soligo-Conegliano, Conegliano, Treviso, Italy; 3 Prevention Department, Local Health Unit 17, Este-Monselice, Este, Padua, Italy; 4 Department of Life, Health and Environmental Sciences, University of L’Aquila, L’Aquila, Italy; 5 Interinstitutional Epidemiology Unit, Local Health Authority, Reggio Emilia, Italy; Arcispedale S. Maria Nuova Research Hospital, IRCCS, Reggio Emilia, Italy; 6 Department of Life, Health and Environmental Sciences, University of L’Aquila, L’Aquila, Italy; Epidemiology and Biostatistics Unit, AUSL Teramo, University of L'Aquila, L'Aquila, Italy; Rudjer Boskovic Institute, CROATIA

## Abstract

**Aim of the study:**

To assess the feasibility of partial HPV genotyping and methylation analysis of *CADM1*, *MAL*, and *miR124-2* genes as triage tests in assaying self-collected cervical samples positive for high-risk HPV on primary screening, and to review the literature regarding host cellular gene methylation analysis of self-collected cervical samples.

**Material and methods:**

Women residing in North-East Italy who had failed to respond to the invitation to participate in an organized population-based program were invited to provide a self-sample. Their stored baseline (self-collected) and follow-up (clinician-collected) cervical samples were included in the study. DNA was extracted from HPV-positive (Qiagen’s Hybrid Capture 2, HC2) samples. Partial genotyping with separate detection of HPV types 16 and 18 was performed with a hybrid capture-based method and a quantitative PCR assay. Methylation was assayed with a quantitative methylation-specific PCR.

**Results:**

High-risk HPV infection was detected in 48% of baseline and 71% of follow-up HC2-positive samples. Methylation was demonstrated respectively in 15% and 23.5% of baseline and follow-up samples and chiefly involved a single gene (*miR124-2*). Invalid quantitative PCR results were recorded in 5% of self-collected samples. The specificity of *miR124-1*, *MAL*, and *CADM1* methylation was 84%, 94%, and 98%, respectively, and the specificity of the three markers combined was 84%. Sensitivity was not estimated due to the lack of CIN2+ samples. The systematic review showed that different methylation assays yield different accuracy values.

**Conclusion:**

Self-collected samples are suitable for methylation assays included in reflex triage testing. The reproducibility and accuracy of the methylation tests described in the literature should be improved.

## Introduction

Cervical screening performed in the framework of organized population-based programs has reduced cervical carcinoma incidence and mortality [1]. Key strategic elements for effective screening include a high coverage of the target population and the use of and adherence to validated protocols.

Cervical cancer is etiologically associated with human papillomavirus (HPV), in particular with persistent infection with high-risk types (hrHPV). Large, randomized, longitudinal clinical trials comparing cytology with hrHPV testing used as primary screening test have clearly demonstrated that HPV DNA testing (where hrHPV types were detected as a pool) was more effective than cytology in reducing the incidence of invasive cancer in women aged 30 years and older [2].

Over the past few years, several trials have explored strategies to increase screening participation and primary screening test performance [3]. In particular, the introduction of the HPV DNA test as the primary assay provides the opportunity to use self-collection devices for cervical samples (a method that is not feasible for the Pap test), whose analytical and clinical accuracy is comparable to that of clinical collection [4]. Furthermore, recent trials [5,6] have demonstrated that home delivery of self-sampling kits increased participation by non-respondents to the standard invitation [7].

A fundamental step of HPV testing strategies is the triage of hrHPV-positive women, to discriminate those at higher risk, who need immediate colposcopy, from those at lower risk, who are invited for re-evaluation (usually at 12 months, using the hrHPV test). Self-collected samples are not suitable for cytology (the most widely used triage test), but can be used in molecular testing [8]. Several molecular assays are being investigated. The most promising results have been obtained with detection of p16 protein (alone or combined with the proliferation marker Ki-67), the identification of HPV types 16 and 18, and the detection of promoter methylation-mediated silencing of the *CADM1* (cell adhesion molecule 1), *MAL* (T-lymphocyte maturation-associated protein), and *miR-124-2* (microRNA-124-2) oncosuppressor genes, which are functionally involved in cervical carcinogenesis [8,9]. HPV types 16 and/or 18 [10] combined with promoter methylation have been associated with a higher risk of high-grade lesion presence and progression [11,12].

In the present study, self-collected samples provided by Italian women who had failed to respond to the invitation to participate in an organized screening program were analysed for HPV DNA. Positive samples were subjected to viral DNA genotyping and tested for the methylation of host cellular genes *CADM1-m18*, *MAL-m1*, and *hsa-miR-124-2*. A systematic review of the literature on the analysis of host cellular gene methylation in self-collected specimens was also performed.

## Material and methods

### Setting

Organized, population-based screening programs of Este-Monselice and Pieve di Soligo Local Health Authorities in Veneto Region (North-East Italy). At the end of 2012, women aged 30–64 years who had failed to respond to the call-and-recall strategy were invited to provide a cervical sample using a second-generation lavage self-sampling device (Delphi Screener, Delphi Bioscience, Scherpenzeel, The Netherlands) delivered at home or picked-up at a reference pharmacy [6]. The self-sampling randomized clinical trial, which was approved by both local Ethics Committees, was registered at www.clinicaltrials.gov (NCT 01647724).

Women with a positive hrHPV test were invited for immediate colposcopy and followed-up according to standard protocols, which involve hrHPV testing of a clinician-collected sample a year later. The subsequent screening round for women who tested negative was at 3 years, either by HPV testing (Este-Monselice) or by cytology (Pieve di Soligo, where it was subsequently implemented with HPV testing).

### Samples

The self-collected samples were delivered to the laboratory, processed as previously described [6], and analysed with the hrHPV Hybrid Capture 2 test (HC2, Qiagen, Hilden, Germany) according to the manufacturer’s instructions [13]. Both baseline and recall specimens were included in this study.

DNA was extracted from all specimens after completion of recall sample collection (i.e. after 16 months for baseline samples and after 1–4 months for recall samples) using Qiagen’s QIAamp DNA mini kit according to the manufacturer’s recommendations, where an aliquot of cells collected before denaturation was resuspended in standard transportation medium (STM, Qiagen), and collected at -80°C.

### HPV genotyping

Partial HPV genotyping was performed on HC2-positive samples using two different assays:

- the HPV Genotyping PS test (Qiagen, based on HC2 technology), where type-specific primers allow individual detection of HPV types 16, 18, 45. The test was performed within 4 months of sample collection by applying the hrHPV HC2 assay procedure to the residual denatured samples; test procedure and data interpretation were according to the manufacturer’s recommendations;
- the HPV-Risk assay (Self-Screen BV, Amsterdam, The Netherlands), a multiplex, real-time PCR-based assay directed against the *E7* gene of 12 hrHPV types (16, 18, 31, 33, 35, 39, 45, 51, 52, 56, 58, 59) and the (probably) high-risk types 66, 67, 68, which uses fluorescent probes to detect PCR products. Four different fluorescent dyes provide for individual detection of HPV 16 and 18, while the Other HPV types are detected as a pool; the internal control for sample adequacy is the human β-globin gene [11]. The assay was performed according to the manufacturer’s instructions using an ABI 7500 Fast Real-time PCR system (Applied Biosystems). A sample was considered invalid when the Ct value of β-globin was < 33 and the HPV 16, HPV 18, and Other HPV type Ct values were > 36 or showed no signal.

A sample was considered positive if it was positive on at least one of the two assays. A concordant result was one where both methods yielded positivity for HPV 16 or 18 (which were individually identified by both assays) or else for type 45 using the PS test and for Other HPV types using the HPV-Risk assay. Samples testing negative on the PS assay and yielding an invalid result on the HPV-Risk assay were considered to be negative for HPV types 16, 18, and 45.

### Methylation analysis

DNA was subjected to bisulphite treatment using the EZ DNA Methylation kit (Zymo Research, Irvine, CA) and tested for methylation of the promoter regions of the cellular genes *CADM1-m18*, *MAL-m1*, and *hsa-miR-124-2* using the PreCursor-M kit (Self-Screen BV, Amsterdam, The Netherlands). This is a quantitative multiplex methylation-specific PCR (qMSP), based on the TaqMan technology. Samples were run in single, separate reactions on an ABI 7500 Fast Real-time PCR system (Applied Biosystems) following the manufacturer’s instructions. According to the manufacturer’s indications, samples with DNA concentrations < 10 ng/μl were excluded from the analysis (n = 3). In each run, 2 additional control samples were included, the HPV 16-positive SiHa cell line and a clinical sample (clinician-collected cervical cells) from a CIN3 case obtained in a previous study [14]. Data analysis and result interpretation were according to the manufacturer’s recommendations. Methylation values comprised between 4 and 9 (well within the allowed 3–12 range) were selected for the baseline start cycle. Different cut-off values were calculated for self-collected samples and clinician-collected scrapes, as indicated by the manufacturer. Samples with a quantification cycle (Cq) value for β-actin > 29 were considered invalid. Samples where the methylation level of at least one of the 3 genes exceeded the predefined ratio described by the manufacturer were considered positive.

### Systematic review

**Fig 1:**
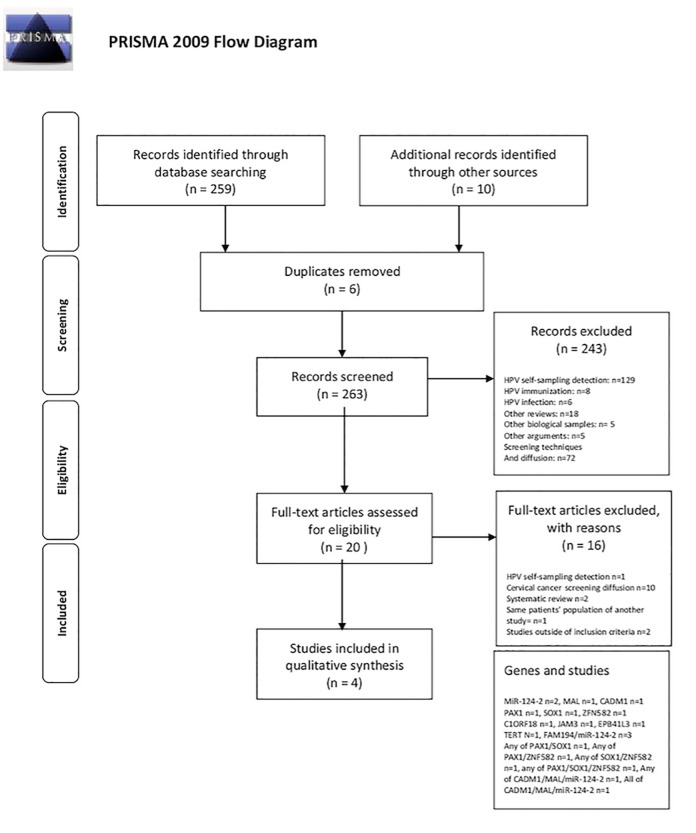
PRISMA Flow-Chart: search strategy.

**Fig 2:**
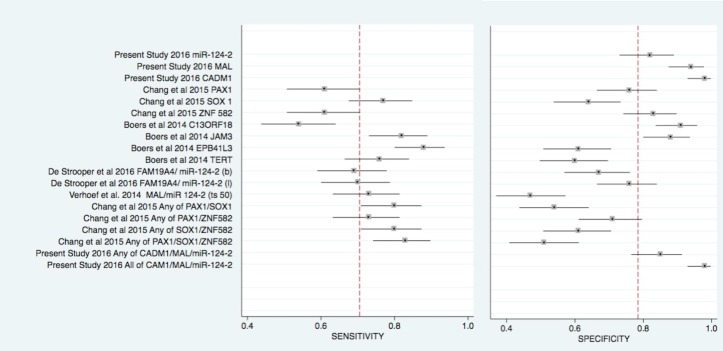
Forrest plot: sensitivity and specificity values of each methylation assay retrieved by the systematic review and of the assay carried out in the present study. Legend: (b): brush; (l): lavage; (ts50) threshold -50 as reported on Ref. [30]

Papers to be included in the systematic review were sought in the MEDLINE, EMBASE, Scopus, Clinicaltrial.gov, Web of Science, and Cochrane Library up to September 2016. The search strategy used the following terms: self-sampling AND cervical cancer; Uterine Cervical Neoplasms AND self-sampling AND methylation; Uterine Cervical Neoplasms AND self-sampling AND gene methylation; cervical cancer AND self-sampling AND methylation; cancer cervix AND self-sampling AND methylation. Papers were selected using the PRISMA flow-chart (Fig 1) and the PRISMA checklist (S1 Table) [15]. A manual search of possible references of interest was also performed. Only studies published in English over the previous 10 years were considered. The papers were selected by two independent reviewers (PMA and ADM); a methodologist (EA) resolved any disagreements. Inclusion criteria were clinical studies and investigations of self-sampling and methylation analysis in women of screening age that reported the specificity and sensitivity of CIN3+ gene markers. Studies of methylation involving physician-collected samples, in vitro studies, and studies validating laboratory techniques were excluded. Bias assessment was performed using the QUADAS-2 scale (S2 Table). The sensitivity and specificity values of each methylation assay, reported in the selected studies, are listed in Fig 2.

## Results

Baseline (self-collected) samples were provided by 454 women, of whom 60 (13.2%) were hrHPV-positive with the HC2 test. Follow-up (clinician-collected) samples were available for 70% (42/60) women, of whom 25 had a negative HC2 test result and 17 (40.5%) had a persistently positive HC2 test result. Their baseline and follow-up data are reported in Table 1.

**Table 1:** Results of high-risk genotyping and methylation analysis of the HPV-positive self-collected baseline samples and clinician-collected follow-up samples.

	Results	BASELINE self-collected samples n = 60	FOLLOW-UP clinician-collected scrapes n = 42
**HPV genotyping No. of samples (%)**			
	Not done	0/60	0/42
	Invalid	0/60	0/42
	Negative	31*/60	25/42
	Positive	29/60 (48.3%)	17/42 (40.5)**; 12/17*** (70.6%)
	HPV 16	0	1
	HPV 16+HR	2	2
	HPV 18	2	0
	HPV 18+HR	0	1
	HR-HPV	25	8
**Methylation analysis No. of samples (%)**			
	Not done	3/60	0/17
	Invalid	3/60	0/17
	Negative	46/54	12/17
	Positive	8/54 (14.8%)	4/17 (23.5%)
	*miR124-2*	5/8	2/4
	*MAL*	0/8	¼
	*MAL+miR124-2*	2/8	¼
	Any of three	1/8	0/4
**Methylation analysis-& HPV genotyping-positive samples (%)**			
		4/54 (7.4%)	3/17 (17.6%)

*29 samples negative on both assays; 2 samples negative on PS and invalid on the HPV-Risk assay, respectively.

** 17/42 persistently hrHPV-positive on the HC2 test.

***11/12 hrHPV-positive cases also at baseline.

### HPV genotyping

Partial genotyping was performed in all baseline and follow-up HC2- positive samples using both the PS test and the HPV-Risk assay (for the latter assay one self-collected sample was not available and could not be tested).

At baseline, hrHPV infection was detected by one or both genotyping assays in 29 cases (48%): HPV 16 in 2 cases, HPV 18 in 2 cases, and Other HPV types in 25 cases, respectively. Two baseline samples that tested negative with the PS assay gave an invalid result on the HPV-Risk assay, whereas a negative result with both assays was recorded in 29 cases (48%).

Genotyping of all 17 HC2-positive follow-up samples was performed with both assays. Infection clearance was documented in 5 women, who at baseline had tested positive for Other hrHPV types. HrHPV infection was detected in 12 cases (71%), a woman whose baseline sample could not be analysed and 11 women who had tested positive at baseline. Among the latter, a concordant result for Other hrHPV types was obtained in 7 cases; HPV 16 was detected in addition to HR in 2 cases and alone in one case, and a mixed infection (HPV 16 at baseline and HPV 18 at follow-up) was found in one case.

### Methylation analysis

SiHa DNA was consistently positive for all 3 markers (median ratio values of 88, 23 and 18 for *CADM1*, *MAL*, and *miR124-2*, respectively), whereas the DNA from the control clinical sample was consistently negative for *CADM1* and positive for *MAL* and *miR124-2* (median ratio values of 13 and 1.3, respectively).

A valid methylation analysis result was obtained in 54/60 (90%) self-collected samples at baseline; 3 samples could not be analysed due to insufficient DNA, and 3 samples gave an invalid result. Overall, DNA methylation of at least one locus (*CADM1*, *MAL*, or *miR124-2*) was detected in 15% (8/54) of the valid samples; 5 samples were positive for a single marker (*miR124-2*), 2 samples were positive for 2 markers (*MAL* and *miR124-2*), and only one was positive for all 3 markers; the latter sample showed the highest ratio values for all markers (0.96, 0.18, 24.5 for *CADM1*, *MAL*, and *miR124-2*, respectively).

Analysis of the methylation markers of all follow-up samples from the 17 HC2-positive women gave valid results. Methylation of at least one locus was detected in 23.5% (4/17) of samples; 3 samples were positive for a single marker (2 for *miR124-2* and one for *MAL*) and one sample was positive for 2 markers (*MAL* and *miR124-2*). *MiR124-2* was the most frequently methylated locus also in follow-up specimens. Its median ratio value in samples positive for a single marker was 1.68. The ratio values of specimens positive for 2 markers were 11.26 and 9.67 (*MAL* and *miR124-2*, respectively). Of the specimens from the 15 women whose baseline and follow-up samples could both be evaluated, 10 were consistently unmethylated, whereas two different methylation patterns were detected in the two specimens in 5 cases: negative / single-positive (*miR124-2*, 2 cases; *MAL*, 1 case), negative / double-positive (*MAL* + *miR124-2*, 1 case), and double-positive (*MAL* + *miR124-2*) / negative (1 case).

Positivity on both methylation analysis and HPV genotyping was documented in 4/54 (7.4%) baseline and 3/17 (17.6%) follow-up samples.

Single-marker specificity ranged from 84% (*miR124-2*) to 98% (*CADM1*). All marker combinations had the same specificity as the least specific test, since the only *CADM1*-positive case also showed methylation of the other 2 loci, and the 3 cases showing MAL methylation also exhibited *miR124-2* methylation (Table 1).

### Systematic review

The database search identified 259 studies and the manual search identified 10, totalling 269 papers on self-sampling. There were 6 duplicates; 243 papers were excluded, because they investigated HPV self-sampling detection (n = 129), HPV immunization (n = 8), HPV infection (n = 6), or described other biological samples (n = 5), were reviews (n = 18), were related to other topics (n = 5), or described screening techniques and screening diffusion (n = 72). Examination of the remaining 20 papers in the second stage of the PRISMA flow-chart led to the exclusion of 16 studies for the following reasons: 1 regarded self-sampling in HPV detection [16], 10 evaluated the diffusion of cervical cancer screening [6,17–25], 2 were systematic reviews [26,27], one examined the same patient population as another study but was the less informative [8], and 2 did not meet the inclusion criteria [12,28] (Fig 1). There remained 4 papers [29–32], which are described in Table 2. The sensitivity and specificity of each gene marker, alone or combined, is reported in Fig 2.

**Table 2:** Characteristics of the four studies included in the systematic review.

Reference year	Sampling methods	Genes	Observational studies	Association and correlation
De Strooper 2016[31]	Cytology + Delphi screener device	• FAM/miR 124–2 • Genotyping HPV16/18	• N = 182 in training set • N = 515 in validation sets	• In validation set for lavage samples: CIN3+ sensitivity of 70.5% (CI95% 60.4–80.6), specificity 67.8% (95% 62.7–73.0%) • In validation set for brush samples: CIN3+ sensitivity of 69.4% (95%CI 58.8–80.1) at a 76.4% (95%CI 70.2–82.6) • In combination with HPV16/18 genotyping, CIN3+ sensitivity and specificity were 88.5% (95%CI: 81.4–95.6) and 46.0% (95%CI 40.4–51.5) for lavage self-samples, and 84.7% (95%CI 76.4–93.0) and 54.9% (95%CI 47.7–62.2) for brush self-samples.
Chang 2015[29]	Cytobrush (Cooper Surgical, CT, USA)	• PAX1 • SOX1 • ZNF582	• Sample size (N) = 136	• PAX1, at cut-off point of 0.014, in self-sampling assays, had sensitivity and specificity of 61.0% (95% CI: 48.0–72.0) and 76% (95% CI 64.0–85.0) • SOX1, at cut-off point of 0.156, in self-sampling assays, had sensitivity and specificity of 77.0% (95% CI: 65.0–72.0) and 76% (95%CI 64.0–85.0) • ZNF582, at cut-off point of 0.214, in self-sampling assays, had sensitivity and specificity of 64.0% (95% CI: 51–75) and 76% (95%CI 77.0–94.0)
			• Normal control = 10	
			• Abnormal cytology = 126 • CIN1 = 37 • CIN2 = 23 • CIN3 = 16 • CIS = 30 • Carcinomas = 20	
Boers 2014[30]	Evalyn Brush (Dry self-sampled)	• C130RF18 • JAM3 • EPB41L • TERT	Sample size (N) = 178	• Methylation levels increased with the severity of the underlying lesion for all genes (p<0.001)
			• Physician taken samples = 128 • Normal cytology = 48 • False negative = 8 • True negative = 40 • Abnormal cytology = 80 • False Positive = 39 • True Positive = 41	
			• Self-sampling = 50 • Histology (CIN3+) 24 • Histology (≤ CIN2) 26	
Verhoef 2014 [32]	Cytology + Delphi screener device	• MAL/ MiR-124-2	• Sample size (N) = 1024 women whit positive self-sampling • 509 –cytology triage - • 515 –methylation triage -	• At Threshold-50, for CIN3+, sensitivity is 73.5 (95% CI 66.3–80.6) and sensibility is 47.2 (95% CI 43.9–50.6 = • At Threshold-50 with HPV16/18 genotyping, sensitivity is 88.4 (95% CI 83.3–93.6) and sensibility is 35.2 (95% CI 32.0–38.4)

## Discussion

In this study, self-collected cervical samples provided by Italian women aged 30 to 64 years who had failed to respond to the invitation to participate in an organized population-based screening program and clinician-collected follow-up samples were subjected to partial HPV genotyping and investigation of the methylation pattern of *CADM1*, *MAL*, and *miR124-2* cellular genes. The present findings confirm the adequacy of self-collected cervical samples to be used in molecular assays suitable for hrHPV triage, even though the absence of high-grade CIN samples in this population limits the significance of our results; in particular, only the specificity of the methylation of our target genes could be calculated.

Similar to a previous study [5], hrHPV infection was detected at baseline in 13% of women (HC2 assay); moreover, infection persistence was found in 40.5% of participants who presented for the follow-up examination at 12 months. These figures are slightly lower than those reported in France (14.3% and 56.6% at baseline 0 and at 12 months, respectively) [33] and are consistent with differences in the populations studied and the HPV assay used.

Partial HPV genotyping with separate identification of types 16 and 18, performed in HC2-positive samples using two different assays, detected an hrHPV type in 48% of the baseline self-collected samples and in 71% of the persistently HC2-positive clinician-collected samples obtained 12 months later. It has been reported [34,35] that HC2 positivity can also result from cross-hybridization with untargeted non-hrHPV types, and that the lower percentage of hrHPV detection by genotyping in self-collected vs. clinician-collected specimens may be due to the presence of vaginal cells containing low-risk HPV types. Indeed, of the 31 women with a negative genotyping result at baseline, 16 were HC2-negative at follow-up, supporting the hypothesis that cross-reactivity with low-risk types yielded some false-positive results at baseline. However, it should be stressed that self-collected specimens cannot be directly compared with those collected by clinicians, which were obtained at a different time point using a different technique.

Partial HPV genotyping has been assessed, alone or in combination with other methods, as an assay to triage hrHPV-positive women [12,36,37]. Since HPV 16 infection has been associated with a cross-sectional and longitudinal higher risk of CIN3+ lesions [15] and HPV 18 infection is associated with adenocarcinoma, both types are of clinical relevance and worthy of individual detection.

Methylation of the cellular genes investigated in the study was detected in a small percentage of self-collected and clinician-collected samples (15% and 23.5%, respectively); these data are consistent with the 19.2% reported by De Strooper et al [31] in self-collected specimens from women without high-grade lesions. Moreover, in more than 60% of samples showing methylation, only one gene (most frequently *miR124-2*) was methylated; in addition, ratio values were considerably lower than those of SiHa cells and the CIN3+ control case, and were lowest in those that were positive for a single gene marker. Methylation of all three genes with high ratio values was recorded only in a 34-year-old woman who moved abroad and was lost to follow-up.

The combination of HPV genotyping and methylation assay data showed a higher frequency of positive results at 12-month follow-up than at baseline (17.6% vs. 7.4%), consistent with a higher rate of persistent HPV infections during follow-up.

In the study, only the specificity of the methylation assay could be analysed due to the absence of CIN2+ or CIN3+ samples, although more than 1000 women were invited to screen with self-sampling.

The specificity values found in this study were much higher than those reported by other studies. In fact, according to the systematic review, the specificity of the *MAL/miR124-2* combination was less than 50% [38]. Clearly, methods and thresholds vary among studies, involving that their results are not directly comparable. Nevertheless, the specificity values found in the present study are the highest reported in relation to any other loci investigated to date. This can be explained by our very low-risk population: in fact, the 1514 self-collected samples tested in the original study (168 of which were HPV-positive) did not include any CIN2+. This surprising finding strongly suggests that the HPV DNA test used in our self-sampling study was endowed with low specificity and yielded a high rate of false-positive results, besides the fact that the population had a low prevalence of lesions. It has clearly been demonstrated that the specificity of a test targeting a risk factor for disease, such as HPV or methylation for CIN2+, is inversely related to disease prevalence [39]; this entails that specificity will be higher in a very low-prevalence than in a high prevalence population.

Finally, as regards the accuracy values of cytology and other candidate triage biomarkers, i.e. p16 and E6-E7 mRNA in clinician-collected samples, sensitivity ranges from 60 to 90% [40] and specificity from 50 to 80%, suggesting that the values measured in self-collected samples need additional evaluations to be better defined.

## Conclusions

Self-collected samples are suitable for methylation assays that could be employed in reflex triage testing. The reproducibility and accuracy of the methylation assays described in the literature needs to be improved before their clinical value can be assessed.

## Supporting information

S1 Table Check-list sections: items according to PRISMA 2009. (DOC)

S2 Table **QUADAS-2:** Risk of Bias assessment according to QUADAS-2 scale available from: (http://www.bristol.ac.uk/social-community-medicine/projects/quadas/quadas-2/ last accessed November 2016).
(DOCX)
