# Supplementary material for: Methylation analysis and HPV genotyping of self-collected cervical samples from women not responding to screening invitation and review of the literature
Source: PLoS One. 2017 Mar 6;12(3):e0172226. doi: 10.1371/journal.pone.0172226 (PMC5338782; doi:10.1371/journal.pone.0172226)
Supplement: S2 Table — QUADAS-2: Risk of Bias assessment according to QUADAS-2 scale available from: (http://www.bristol.ac.uk/social-community-medicine/projects/quadas/quadas-2/ last accessed November 2016). (DOCX) [file pone.0172226.s002.docx]

| **Study** | **RISK OF BIAS** | | | | **APPLICABILITY CONCERNS** | | |
| --- | --- | --- | --- | --- | --- | --- | --- |
|  | **PATIENT SELECTION** | **INDEX TEST** | **REFERENCE STANDARD** | **FLOW AND TIMING** | **PATIENT SELECTION** | **INDEX TEST** | **REFERENCE STANDARD** |
| De Strooper | ☺ | ☺ | ? | ☺ | ☺ | ☺ | ☺ |
| Chang | ☺ | ☺ | ? | ☺ | ☺ | ☺ | ☺ |
| Boers | ☺ | ☺ | ? | ☺ | ☺ | ☺ | ☺ |
| Verhoef | ☺ | ☺ | ? | ☺ | ☺ | ☺ | ☺ |

☺Low Risk ☹High Risk ? Unclear Risk

Table S2: Risk of Bias assessment according to QUADAS-2 scale available from: (<http://www.bristol.ac.uk/social-community-medicine/projects/quadas/quadas-2/> last accessed November 2016)
